# Did Tenascin-C Co-Evolve With the General Immune System of Vertebrates?

**DOI:** 10.3389/fimmu.2021.663902

**Published:** 2021-04-12

**Authors:** Gertraud Orend, Richard P. Tucker

**Affiliations:** ^1^ Inserm U1109, The Tumor Microenvironment Laboratory, INSERM UMR_S 1109, Faculté de Médecine, Hopital Civil, Institut d’Hématologie et d’Immunologie, Strasbourg, France; ^2^ Université Strasbourg, Strasbourg, France; ^3^ Fédération de Médecine Translationnelle de Strasbourg (FMTS), Strasbourg, France; ^4^ Department of Cell Biology and Human Anatomy, University of California at Davis, Davis, CA, United States

**Keywords:** tenascin, immunity, evolution, phylogeny, chemokine, development

## Abstract

Tenascin-C plays important roles in immunity. Toll-like receptor 4, integrin α9β1 and chemokines have already been identified as key players in executing the immune regulatory functions of tenascin-C. Tenascin-C is also found in reticular fibers in lymphoid tissues, which are major sites involved in the regulation of adaptive immunity. Did the “tool box” for reading and interpreting the immune-regulating instructions imposed by tenascins and tenascin-C co-evolve? Though the extracellular matrix is ancient, tenascins evolved relatively recently. Tenascin-like genes are first encountered in cephalochordates and urochordates, which are widely accepted as the early branching chordate lineages. Vertebrates lacking jaws like the lamprey have tenascins, but a tenascin gene that clusters in the tenascin-C clade first appears in cartilaginous fish. Adaptive immunity apparently evolved independently in jawless and jawed vertebrates, with the former using variable lymphocyte receptors for antigen recognition, and the latter using immunoglobulins. Thus, while tenascins predate the appearance of adaptive immunity, the first tenascin-C appears to have evolved in the first organisms with immunoglobulin-based adaptive immunity. While a C-X-C chemokine is present in the lamprey, C-C chemokines also appear in the first organisms with immunoglobulin-based adaptive immunity, as does the major histocompatibility complex, T-cell receptors, Toll-like receptor 4 and integrin α9β1. Given the importance of tenascin-C in inflammatory events, the co-evolution of tenascin-C and key elements of adaptive and innate immunity is suggestive of a fundamental role for this extracellular matrix glycoprotein in the immune response of jawed vertebrates.

## Introduction

Tenascins are extracellular matrix glycoproteins with one or more epidermal growth factor-like repeats, multiple fibronectin type III (FNIII) domains, and a C-terminal fibrinogen-related domain (FReD) (1). In bony fishes and tetrapods there are four tenascins. The first tenascin to be discovered and characterized was tenascin-C (2), which is widely expressed in the embryo at sites of cell motility and other forms of active morphogenesis but has a much more restricted distribution in adult organisms (3). Tenascin-R (4) and tenascin-W (5, 6) are primarily expressed in the developing nervous system and in developing bone, respectively, though tenascin-W is also found together with tenascin-C in certain stem cell niches in the adult (7). Tenascin-X is widely expressed in loose connective tissue during development and in the adult (8).

In addition to expression in the embryo, tenascin-C is expressed in the adult in a variety of pathologic situations, notably in the stroma of most solid tumors (9) and at other sites of inflammation (10). Midwood et al. (11) found that chronic inflammation associated with rheumatoid arthritis (RA) requires the expression of tenascin-C, and that joint damage from induced erosive arthritis is limited in mice lacking tenascin-C. These authors went on to show that tenascin-C’s FReD is a ligand for Toll-like receptor 4 (TLR4), and that tenascin-C acts through TLR4-mediated signaling to initiate the production of pro-inflammatory cytokines (11). Tenascin-C is also an integrin ligand (12), and through integrin α9β1 tenascin-C can induce the expression of pro-inflammatory chemokines such as CCL2, CCL4 and CXCL5 (13). Correspondingly, the expression of CXCL2 is reduced in the absence of tenascin-C in an animal model of liver ischemia and reperfusion injury (14). Using a murine RA model of joint injury, Ruhmann et al. (15) showed that tenascin-C plays an active role in the polarization of Th17 lymphocytes, demonstrating a role for tenascin-C in inflammatory damage from the adaptive immune system. Tenascin-C can promote cancer progression in many ways (9, 16, 17). Recently tenascin-C was shown to contribute to the immune-suppressive microenvironment of the tumor stroma through integrin α9β1 inducing CCL21 (in lymphatic endothelial cells) and TLR4 regulating CCR7 (in CD11c+/dendritic cells) (18, 19). This suggests that cancer cells may be able to hijack important immune-related functions of tenascin-C in tumors.

In this mini review we will explore the possibility that tenascin-C appeared during evolution along with other critical players in the immune system, pointing to fundamental roles for this extracellular matrix glycoprotein in regulating inflammatory events. We will also consider the possibility that tenascin-C acts through some of the same players to perform similar roles during embryonic development. 

## The Evolution of Tenascins

**Figure 1:**
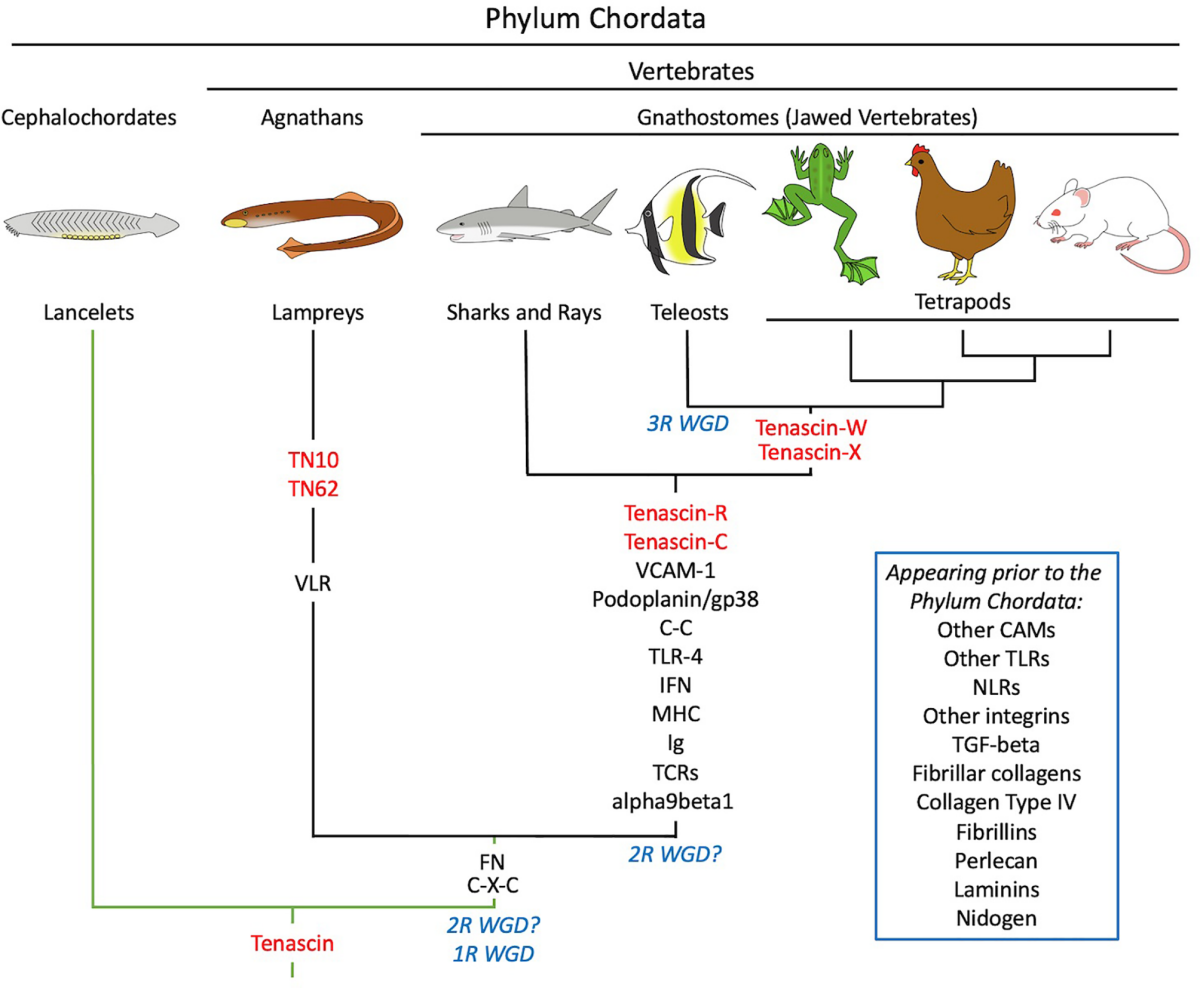
A schematic illustration of the co-evolution of tenascins (in red) and elements of the immune systems of representative chordates. Tenascins first appeared in invertebrate members of the phylum Chordata like the cephalochordates. Branches in green indicate chordates lacking an adaptive immune system, while branches in black indicate chordates with both innate and adaptive immunity. In the vertebrates, the jawless agnathans (e.g., lamprey) evolved adaptive immunity based on variable lymphocyte receptors (VLRs); the first chemokines (C-X-C) and fibronectin (FN) appeared at this time as well. Tenascin-C first appeared in the jawed vertebrates (gnathostomes), together with immunoglobulin (Ig)-based adaptive immunity, the major histocompatibility complex (MHC), additional chemokines (C-C), interferons (IFN), T-cell receptors (TCR), Toll-like receptor 4 (TLR4) and the integrin α9β1. Podoplanin/gp38 and VCAM-1 appeared at this time as well. Many key elements of reticular fibers and vertebrate immune systems predate the evolution of chordates and are included in the inset. See text for details. 1R WGD, First round whole genome duplication; 2R WGD (with the question marks showing two proposed periods for this event), Second round whole genome duplication; 3R WGD, third round whole genome duplication; CAMs, cell adhesion molecules; TN10 and TN62, lamprey-specific tenascins; NLRs, nucleotide-binding oligomerization domain-like receptors.

Phylogenetic analysis can be used to predict the first appearance of a protein during evolution, and in turn this can be used to infer an explanation for the evolution of the protein. Some well-studied extracellular matrix genes encoding components like fibrillar collagens, laminins and thrombospondins are found in the genomes of sponges and sea anemones, indicating that they evolved prior to specialized connective tissues and a complex nervous system (20). Tenascins, in contrast, are not found in the genomes of animals outside the phylum Chordata (21, 22). Invertebrate members of the phylum like the cephalochordates and urochordates have a single tenascin gene (i.e., prior to the whole genome duplication events of ancestral vertebrates), but when included in the construction of phylogenetic trees these tenascins do not belong to any of the four tetrapod tenascin clades. Two tenascins are found in the genome of the Japanese lamprey *Lethenteron japonicum* and one in the genome of the sea lamprey *Petromyzon marinus*. But like the tenascins from invertebrates, the tenascins from these jawless (agnathan) fish do not sort to the tenascin-C, -R, -W or -X clades. Cartilaginous fish like the elephant shark *Chalorhincus milii*, in contrast, have tenascin-C and tenascin-R, while bony fish and tetrapods have all four tenascin paralogs (23). Thus, tenascin-C and tenascin-R evolved together with the first jawed vertebrates (gnathostomes), and additional members of the family appeared later during evolution. The evolution of tenascins is summarized in **Figure 1**. 

## The Evolution of Adaptive Immunity

Like most protostomes and echinoderms, cephalochordates have an extremely complicated innate immune system. An early analysis of the *Branchiostoma* genome revealed 48 TLR genes and 92 nucleotide-binding oligomerization domain-like receptors, among hundreds of other genes related to innate immunity (24). A more recent examination revealed 30 additional TLRs and confirmed their expression (25). However, cephalochordates lack an adaptive immune system. In contrast, jawless vertebrates like the lamprey were first shown, over half a century ago, to have both innate and adaptive immune systems (26). The lamprey’s adaptive immune system is based not on recombination-activating gene-mediated rearrangement of light chains and heavy chains to make immunoglobulins, but instead on rearrangement based on leucine-rich repeat cassettes to create variable lymphocyte receptors (VLRs) (27). The lamprey’s immune cells express one VLR per cell, and secreted VLR dimers form pentamers (28), not unlike IgM. These stunning examples of convergent evolution were recently reviewed by Flajnik (29). In contrast, all jawed vertebrates, from cartilaginous fish to mammals and birds, have an adaptive immune system based on immunoglobulins, T-cell receptors, and the major histocompatibility complex (MHC). The evolution of immunoglobulin-based adaptive immunity in gnathostomes has been thoroughly reviewed by others (30–34). Thus, adaptive immunity is seen in all vertebrates, but it has evolved independently into a VLR-based system in jawless vertebrates, and into an immunoglobulin-based system in jawed vertebrates. 

## The Evolution of Chemokines

Chemokines are secreted factors that influence cell motility both in the embryo and in the immune system. They are classified according to the arrangement of cysteine residues found at the amino terminus of the protein (C-C, C-X-C, CX3C and XC). Their receptors are named using the same schema (CCR, CXCR, CX3CR and XCR) (35). Invertebrates, including cephalochordates and urochordates, do not have chemokines (24), but a C-X-C chemokine (an IL-8 homologue) has been found in the lamprey (36), and extensive analysis of the lamprey genome reveals three CXCRs homologous to CXCRs from human (37). The same study demonstrated the presence of 6 CCRs, 5 CXCRs and a XCR in the elephant shark, and even more in bony fishes. Thus, while C-X-C chemokines evolved with the first vertebrates, the large number and diversity of chemokines found in mammalian genomes first appeared with the evolution of jawed vertebrates.

## The Evolution of the Extracellular Matrix of Reticular Fibers

The presence of tenascin-C in the reticular fibers of lymphoid organs (38) and in tumor matrix tracks (39) is remarkable and may represent an ancient defense program that is reused, or perhaps better characterized as mis-used, in cancer. However, most of the other extracellular matrix molecules found in reticular fibers appear to be more ancient than tenascin-C. For example, fibrillar collagens, collagen type IV, fibrillins, perlecan, laminins and nidogen are found in the genomes of sponges and placozoans (20). Other specific collagen types found in reticular fibers appear significantly later, but still predate the jawed vertebrates (e.g., collagen type XII is found in urochordates [Gene ID 100182938]). Fibronectin evolved in jawless vertebrates (23), i.e., after tenascins but before tenascin-C and gnathostome-specific immunity. Fibroblast reticular cells (FRCs) are an important stromal cell type that shapes the structure and function of lymph nodes (40). FRC markers such as podoplanin/gp38 and VCAM-1 appear to have co-evolved with tenascin-C in jawed fishes (41) (XM_033029124.1), though other cell adhesion molecules are quite ancient (42). Remarkably, stroma in oral squamous cell carcinomas has lymphoid properties characterized by abundant FRCs expressing extracellular matrix components of lymph nodes including tenascin-C and utilizing CCR7/CCL21 signaling for retaining CD11c+ immune cells in the tumor matrix tracks. Moreover, in the absence of tenascin-C these lymphoid properties are largely diminished suggestive of tenascin-C as an orchestrator of these tissues (19).

## Discussion

Tenascins appeared with the first chordates, but tenascin-C evolved with the jawed vertebrates. This coincides with the evolution of immunoglobulin-based adaptive immunity, the MHC, most chemokines, T-cell receptors, interferon Types I and II (43), and TLR-4 (**Figure 1**). Given the recently identified roles of tenascin-C in regulating inflammatory events, tenascin-C may have evolved, in part, to play a key function in adaptive and innate immunity in jawed vertebrates. The high amino acid sequence conservation in tenascin-C (44) and the absence of gross deletions of tenascin-C underscores a potential important role in the organism, perhaps related to fine-tuning adaptive immunity. Interestingly, α9β1 integrin also evolved in vertebrates even though homologues of other alpha integrin subunits are found much earlier in sponges and sea anemones (45, 46).

Not all of the hardware in the mammalian immunoglobulin-based adaptive immunity tool kit co-evolved with tenascin-C in cartilaginous fishes. As described above, some chemokines predate the appearance of tenascin-C, and other key elements appear to have evolved after. For example, the natural killer cell activating receptor NKG2D is not found in fishes, amphibians, reptiles or birds, but is limited instead to mammals, including monotremes (e.g., the platypus) (XM_029081597.1). Others, like transforming growth factor β, appeared earlier in the first deuterostomes (47).

Mucosal fluids such as breast milk have anti-HIV activity, and this activity is mimicked with exogenous tenascin-C and lost when naturally occurring tenascin-C is removed from breast milk (48). Tenascin-C is proposed to block the interaction between the HIV-1 envelope protein (Env) and the coreceptor CCR5/CXCR4 *via* binding to the HIV-1 Env V3 loop *via* the FNIII and FReD domains in tenascin-C, and appears to require oligomerization and N-linked glycosylations (49). Thus, tenascin-C can also play an important role in preventing infection through pathways independent of the traditional innate and adaptive immune systems. This may be another reason why the tenascin-C sequence is so highly conserved.

While tenascin-C is expressed during inflammation, it is also abundant in the normal embryo. For example, tenascin-C is found in the extracellular matrix surrounding neural crest cells (50), a population of migratory cells that also appears to have evolved in the first vertebrates (51). Neural crest cells themselves make tenascin-C (52), and they stop migrating if this tenascin-C expression is disrupted with antisense morpholinos (53). Tenascin-C may have similar roles during inflammation and development. For example, neural crest cell migration into the pharyngeal arches of the chicken embryo is disrupted by CXCR4 antagonists (54), and CXCR4-null mice have abnormally small dorsal root ganglia, which are formed from the neural crest (55). As the CXCR4 ligand, SDF-1/CXCL12, is also a chemoattractant for T-lymphocytes (56), tenascin-C may be acting through similar pathways in the embryo and during the immune response.

One of the places where tenascin-C is expressed in the adult organism, and in the embryo, is in stem cell niches (e.g., neural, hair follicle, dental pulp, periosteal, hematopoietic and lymphoid progenitor stem cell niches) (7). As in immunity, the many roles of chemokines in a variety of stem cell niches in regulating cell proliferation and migration are well known, suggesting the use of similar tool kits in diverse systems (57–59). Future studies can be directed toward exploring potential roles for tenascin-C and chemokine expression and functions in the stem cell niches.

What does phylogenetic analysis tell us about tenascin-C and its role as a TLR-4 ligand? While TLRs are ancient parts of the innate immune system that predate the evolution of tenascins by hundreds of millions of years (60–62), TLR-4 is a relatively new member of the family that co-evolved with tenascin-C in jawed vertebrates (63). However, interactions between the FReD of tenascin-C and TLR-4 may not be limited to this member of the tenascin family, as the binding pocket of the FReD is found in the other tenascin family members as well (64). One intriguing possibility is that the tenascin/TLR interactions may have predated the roles currently being found for tenascin-C in the immune systems of vertebrates and may indicate a fundamental role for tenascins in invertebrate chordates in regulating their innate immunity. Future studies should address in more detail the common determinators of how tenascin-C regulates innate and adaptive immunity through TRL4, integrin α9β1, chemokines and other yet-to-be-identified partners. This could also be important in the defense against microbes, as described above with HIV-1.

We have focused here on well-known elements of innate and adaptive immunity in vertebrates and especially on molecules with known connections to tenascin-C; future studies should concentrate on other players in the context of the evolution of extracellular matrix.

## Author Contributions

GO conceived the review topic and contributed to the manuscript’s outline, editing, and literature search. RPT wrote the manuscript and prepared the figure. All authors contributed to the article and approved the submitted version.

## Funding

GO is supported by INSERM, the University Strasbourg, Agence National de la Recherche (ANR-ACKITEC, ANR-MatrixNash), Institut National Contre le Cancer (INCa PLBIO-TENMAX), and L’Alliance nationale pour les sciences de la vie et de la santé (AVIESAN-Radio3R).

## Conflict of Interest

The authors declare that the research was conducted in the absence of any commercial or financial relationships that could be construed as a potential conflict of interest.
